# Dynamical origin of near- and below-threshold harmonic generation of Cs in an intense mid-infrared laser field

**DOI:** 10.1038/ncomms8178

**Published:** 2015-05-20

**Authors:** Peng-Cheng Li, Yae-Lin Sheu, Cecil Laughlin, Shih-I Chu

**Affiliations:** 1 Center for Quantum Science and Engineering, and Center for Advanced Study in Theoretical Sciences, Department of Physics, National Taiwan University, Taipei 10617, Taiwan; 2 College of Physics and Electronic Engineering, Northwest Normal University, Lanzhou 730070, China; 3 Department of Chemistry, University of Kansas, Lawrence, Kansas 66045, USA; 4 School of Mathematical Science, University of Nottingham, Nottingham NG7 2RD, England

## Abstract

Near- and below-threshold harmonic generation provides a potential approach to generate vacuum-ultraviolet frequency comb. However, the dynamical origin of in these lower harmonics is less understood and largely unexplored. Here we perform an *ab initio* quantum study of the near- and below-threshold harmonic generation of caesium (Cs) atoms in an intense 3,600-nm mid-infrared laser field. Combining with a synchrosqueezing transform of the quantum time-frequency spectrum and an extended semiclassical analysis, the roles of multiphoton and multiple rescattering trajectories on the near- and below-threshold harmonic generation processes are clarified. We find that the multiphoton-dominated trajectories only involve the electrons scattered off the higher part of the combined atom-field potential followed by the absorption of many photons in near- and below-threshold regime. Furthermore, only the near-resonant below-threshold harmonic is exclusive to exhibit phase locked features. Our results shed light on the dynamic origin of the near- and below-threshold harmonic generation.

## Abstract (web summary)

High harmonic generation (HHG) above the HHG threshold is understood using the three-step model, but the near- and below-threshold regimes are largely unexplored. Here, Li *et al*. shed light on the dynamic origin of the near- and below-threshold harmonic generation of caesium atoms in an intense mid-infrared laser field.

## Graphical abstract

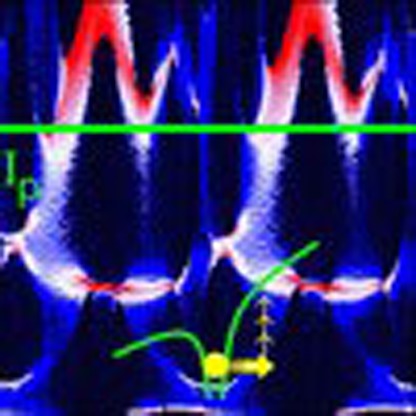

High-order harmonic generation (HHG) of atoms and molecules in the intense laser fields render technologies for the extreme-ultraviolet and soft-X-ray sources on the attosecond time scale, leading to applications such as the observation and control of the electronic dynamic behaviours^1^,^2^,^3^,^4^,^5^,^6^,^7^,^8^. The HHG spectrum is characterized by a rapid drop at low orders followed by a broad plateau where all harmonics have similar amplitudes and finally a sharp cutoff beyond which no further harmonic emission is seen. The cutoff of the HHG is located approximately at the energy *I*_*p*_+3.17*U*_*p*_, where *I*_*p*_ is the atomic ionization potential and *U*_*p*_ is the ponderomotive potential. The HHG process for the harmonics above the ionization threshold *I*_*p*_ can be well understood by the semiclassical three-step model^9^,^10^ and the strong field approximation^11^. In the framework of the three-step model, the bound electron is first freed by either tunnelling or multiphoton ionization and then accelerated in the applied laser field. In the last step, depending on the initial time and the laser profile, the electron can be driven back towards the parent core to recombine into the ground state, generating harmonics in this process. The electron trajectories fall into two families, namely short and long trajectories, depending on the ionization and return time of the electrons.

In the past, the major attention was focused on the regime above the threshold. More recently considerable attention has been paid to the near- and below-threshold regime^12^,^13^,^14^,^15^,^16^,^17^,^18^,^19^,^20^ as a potential source of coherent vacuum-ultraviolet radiation^7^. While the semiclassical three-step model and the strong field approximation are effective to explain the process for the above-threshold harmonics, neglecting the atomic interaction potential in both the models results in inadequate description of the process in the near- and below-threshold harmonic generation. Nevertheless, experimental observations have shed some light on the mechanism for the near- and below-threshold regime. Power *et al*.^12^ and Yost *et al*.^13^ suggest that the generation of the below-threshold harmonics is dominated by long trajectories, owing to the influence of the atomic potential. Hostetter *et al*.^14^ extend the semiclassical three-step model with the atomic potential for the treatment of below-threshold harmonic generation processes in a model atom. By studying near-threshold HHG from the aligned molecules, Soifer *et al*.^15^ show that near-threshold long trajectories belong to the conventional three-step model while the short trajectories stem from multiphoton-driven pathways. More recently, Chini *et al*.^16^ have shown that the phase-matched below-threshold harmonics are generated only near the resonance structures of the atomic target.

In this work, we present an *ab initio* study of the near- and below-threshold harmonic generation of caesium (Cs) atom in an intense mid-infrared laser field by solving the three-dimensional time-dependent Schrödinger equation (TDSE) accurately and efficiently by means of the time-dependent generalized pseudospectral method^21^. For a proper account of the low-lying and Rydberg states of Cs, an accurate angular momentum-dependent model potential is constructed, taking into account the electronic structures of the ground and exited states as well as the oscillator strengths. In addition, we employ a synchrosqueezing transform (SST)^22^,^23^,^24^,^25^ (Supplementary Method) to analyse the time-frequency spectra of the near- and below-threshold HHG of Cs. By comparing the SST time-frequency spectra and the extended semiclassical calculations, we distinguish the contributions of the short trajectories, long trajectories, multi-rescattering trajectories and resonant trajectories in the near- and below-threshold harmonic generation, and demonstrate the features of the spectral dynamical phase. We find that the multiphoton-dominated processes only occur when the electrons are scattered off the higher part of the combined atom-field barrier potential (HBP) followed by the absorption of many photons in near- and below-threshold harmonic generation. In particular, we uncover a type of trajectory near the resonant harmonic in the below-threshold generation, where the phase at the peak intensity is constant for every optical cycle .These resonant trajectories are only the type that have locked dynamical phases. Furthermore, the frequency comb is produced by superposing several below-threshold harmonics of Cs, and the result is in good agreement with the recent experimental results^12^.

## Results

### Harmonic spectra of Cs

**Figure 1:**
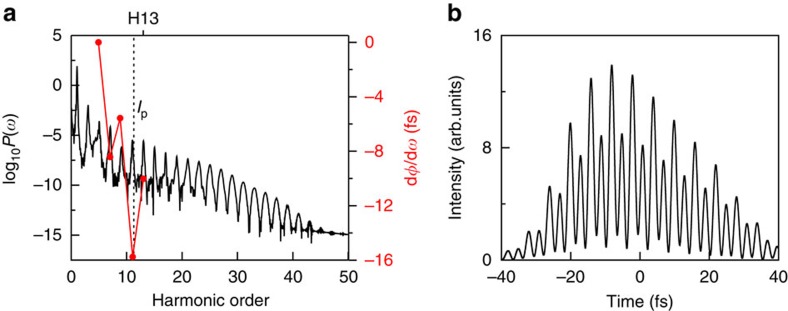
HHG power spectra of Cs and time-domain synthesis of the harmonics. (**a**) The HHG power spectrum of Cs driven by an intense 3,600-nm (mid-infrared) laser pulse with the peak intensity *I*=1.4 × 10^12^W cm^−2^. The black vertical dashed line indicates the corresponding ionization threshold marked by *I*_*p*_. The d*φ*/d*ω* denotes the emission time associated a group of harmonics with the central frequency *ω*, where *φ*(*ω*) is the spectral phase of the HHG. (**b**) A time-domain superposition of the harmonics 7–13. Note that the maximum burst has a duration of about 4.3 fs.

**Figure 2:**
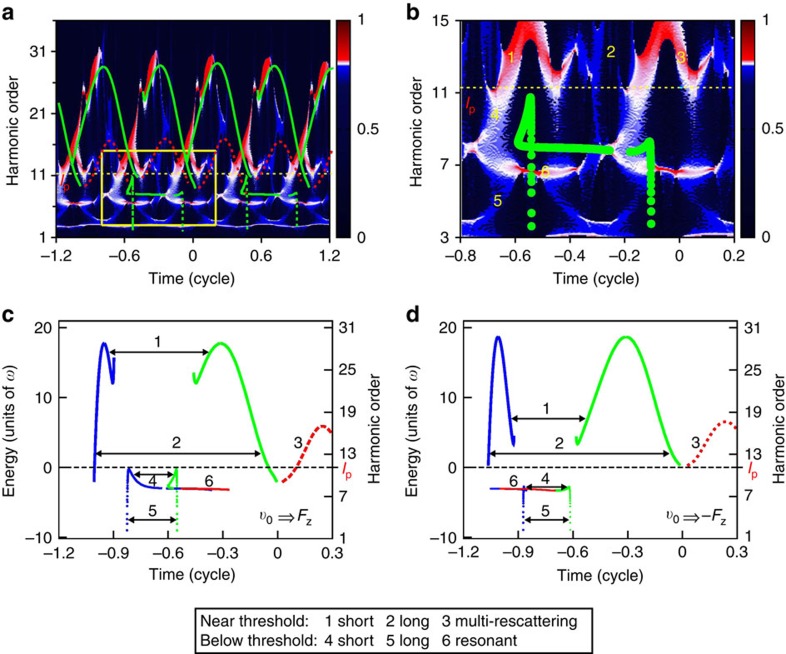
SST time-frequency spectra and semiclassical trajectories. (**a**) SST time-frequency analysis of the HHG spectra of Cs. For comparison, the green curves (both solid lines and dots) and the red dashed lines indicate the semiclassical trajectories of the first return and second return, respectively. (**b**) An enlarged view of the SST time-frequency profiles in the near- and below-threshold regions (marked by the yellow window in **a**). (**c**) Semiclassical return energy as a function of ionization time (blue lines) and return time (green lines and red dashed lines). Here the initial condition is that the electrons with an initial velocity *v*_0_ are released along the electronic-field force **F**_*z*_. For clarity, we only show the semiclassical simulation for the electrons released during one optical cycle preceding the pulse peak. (**d**) Same as **c**, for the electrons with an initial velocity *v*_0_ are released against the electronic-field force **F**_*z*_. The laser parameters used are the same as those in Fig. 1a.

Figure 1a shows the HHG power spectrum of Cs atom calculated by solving the three-dimensional TDSE with an accurate angular momentum-dependent model potential in an intense mid-infrared laser field (Supplementary Method and Supplementary Fig.1). In the calculation, we adopt a 3,600-nm mid-infrared laser wavelength^12^, with a cosine-squared shape and a duration of 20 optical cycles (the full width at half-maximum pulse duration is about 87 fs) and the intensity is *I*=1.4 × 10^12^ Wcm^−2^. The corresponding Keldysh parameter *γ* is equal to 1.1 (
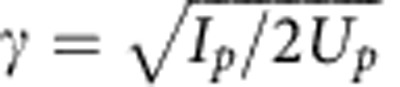
), which indicates an intermediate ionization regime (multiphoton ionization is typically selected by *γ*≫1 while tunnelling ionization is by *γ*≪1). The atomic ionization potential of Cs is equal to 0.143099, a.u. (Supplementary Table 1), which coincides with the 11.3 harmonic order. The black vertical dashed line shown in Fig. 1a indicates the corresponding ionization threshold marked by *I*_*p*_. For the 3,600-nm laser wavelength, the harmonic 13 (H13) is just above the threshold. In Fig. 1a, d*φ*/d*ω* represents the emission time associated with a group of harmonics with the central frequency ω, where *φ*(*ω*) is the spectral phase of the HHG. The d*φ*/d*ω* (harmonics 5–13) shows a negative trend as the harmonics shift from the below-threshold to the near-threshold regions, implying the tunnelling ionization becomes the dominant mechanism. Furthermore, we show that a maximum burst of 4.3 fs synthesized by the harmonics 7–13 in Fig. 2b. This result is in good agreement with the experimental results^12^, indicating that the superposition of the below-threshold HHG provides a potential method to produce the frequency comb.

### SST time-frequency analysis and semiclassical trajectories

To analyse the underlying mechanism from the *ab initio* simulation, we perform the time-frequency analysis on the induced dipole moment of Cs atom interacting with the applied laser field. In our previous study^22^, several representative time-frequency methods have been compared for the hydrogen system, including the Gabor transform, the Morlet transform, the Wigner–Ville transform^23^ and SST, as examples for the short-time Fourier transform, as well as the continuous wavelet transform, the bilinear time-frequency transform and the reallocation method, respectively. We found that both the Gabor and the Morlet transforms are subject to some obscure spectral features arising from a window and that the Wigner–Ville transform is accompanied by interference artifacts, resulting in incomprehensible analysis. Among these methods, only the SST can resolve the intrinsic blurring in the the Gabor and the Morlet transforms^24^. As a result, we adopt the SST to explore the characteristic behaviours of harmonic spectra below the ionization threshold, which has successfully depicted chronotaxic systems^25^ and cardiovascular systems^26^.

The time-frequency representation in Fig. 2a shows a periodic repetition of arches comprising the short and long trajectories. It is readily observed that the main contribution to the above-threshold harmonics is due to the short trajectories. To explore the intricate structures in the near- and below-threshold generation, an enlarged view is displayed in Fig. 2b. In this figure, the trajectories that lie between the harmonic 9 and 13 suggest multiple returns of the electron, and the prominent trajectory located near the vicinity of the 7th harmonic could be the 6*s*–7*p* multiphoton resonance-transition of Cs. To explore the dynamical role of the quantum trajectories, we extend a standard semiclassical approach suggested independently by Corkum^9^ and Kulander *et al*.^10^ with the inclusion of the atomic potential. Here the electric-field force corresponding to the applied laser field in atomic units is **F**_*z*_=−*E*(*t*)**e**_*z*_, where **e**_*z*_ is the unit vector in the *z* direction and *E*(*t*) is the electric field of the laser pulse. For the laser parameters used, the corresponding Keldysh parameter *γ* is >1 and the multiphoton ionization regime is expected to be dominant, the initial conditions are provided by releasing the electrons with an initial velocity to overcome a potential barrier. Therefore, the direction of the electron initial velocity is either ‘identical' or ‘opposite' with respect to **F**_*z*_. Note that when the direction of the electron initial velocity is ‘identical', the electron gains an extra energy to escape the barrier; when the direction of the electron initial velocity is ‘opposite', the electron is pushed back when leaving the barrier. The semiclassical return energy as a function of the ionization time and return time of the electrons that are released in the first one cycle before the pulse peak for ‘identical' and ‘opposite' conditions are presented in Fig. 2c,d, respectively. We can indicate the short trajectories 1 and long trajectories 2 as those in the standard three-step model, as well as the multi-rescattering trajectories 3 (second return). The below-threshold trajectories that released late and return early are regarded as the below-threshold short trajectories 4, while those released early and return late are the below-threshold long trajectories 5. Furthermore, the trajectories marked by 6, which are released and return almost immediately, are named as the resonant trajectories, due to that they only contribute to the near-resonance harmonic, that is, the 7th harmonic order, which coincides with the 6*s*–7*p* multiphoton resonance-transition of Cs. Note that the resonant trajectories are relatively insensitive to the ‘identical' and ‘opposite' initial conditions. The trajectories 1, 2, 3, 4, 5 and 6 are superimposed in Fig. 2a,b for the sake of comparison with the SST representation. As shown in the figures, the structures of the SST representation are in good agreement with the semiclassical trajectories. Trajectories with ‘identical' and ‘opposite' initial conditions interchange because the laser field changes sign every half optical cycle. In the SST representation, trajectories with both ‘identical' and ‘opposite' initial conditions are appear in one optical cycle.

The contribution of quantum trajectories to the near- and below-threshold harmonic generation can be understood according to Fig. 2b. For the near-threshold harmonics 11–13, the short trajectories 1 and the multi-rescattering trajectories 3 have the major contributions, while the long trajectories 2 contribute little. As Power *et al*.^12^ point out that long trajectories are the favoured pathways in near- and below-threshold harmonic generation, our simulation and time-frequency analysis results further indicate that these long trajectories are the multi-rescattering trajectories. In the below-threshold region, the short trajectories 4 mainly contribute to harmonics 9–11, while the long trajectories 5 contribute to harmonics 3–5. Note that the below-threshold short trajectories 4 for harmonics 9–11 are resulted from the ‘identical' initial condition. As for the resonance harmonic, that is, the 7th harmonic, the dominant trajectories are the resonant trajectories 6. Note that the intensity of the time-frequency representation is strong due to the enhancement of the resonance. The strongest resonant emissions for each optical cycles are located at near the laser peak intensity.

### Electron dynamics in near- and below-threshold HHG

**Figure 3:**
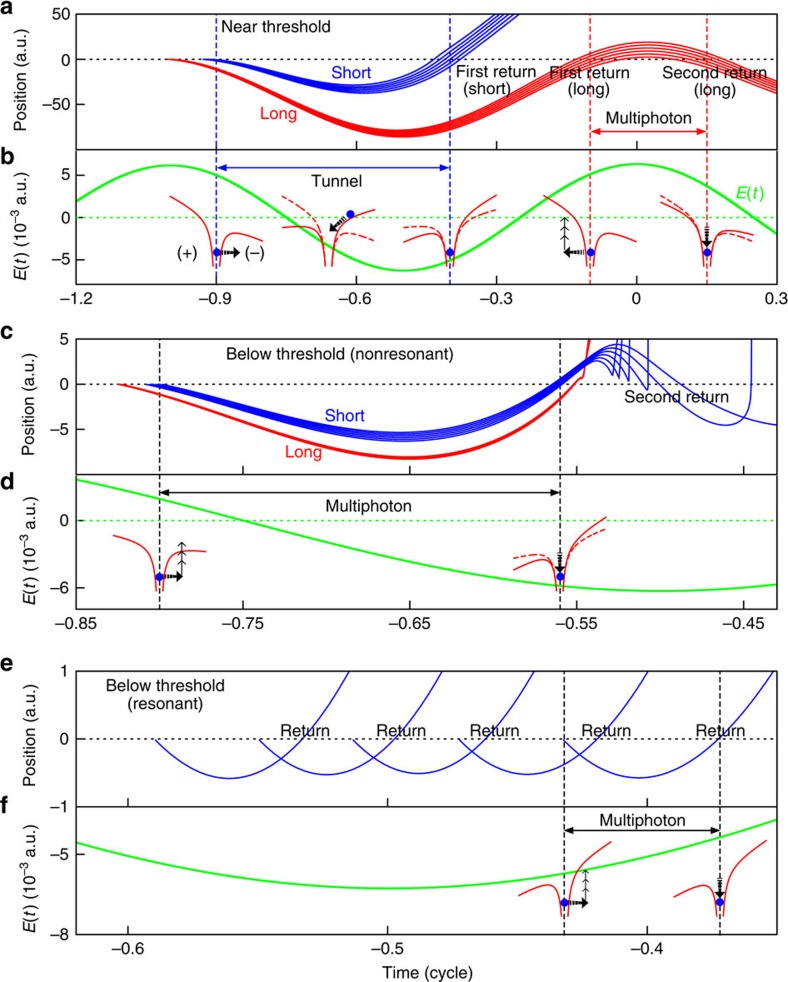
Position and time, and scheme of electron dynamics. (**a**) Several short trajectories and long trajectories (first return and second return) in near-threshold HHG. (**b**)Time-dependent laser fields *E*(*t*; green solid line) and the illustration for the corresponding electron dynamics (red lines), which indicates that the tunnelling regime is dominant for the first return, while multiphoton regime is dominant for the second return. (**c**) Several short trajectories and long trajectories in below-threshold HHG (nonresonant). (**d**) The illustration of the electron dynamics (red lines) in the laser field, indicating that the electron behaviour is in the multiphoton regime. (**e**) Same as **c**, for below-threshold HHG (resonant). (**f**) The laser field and the illustration sketch of the corresponding scheme of electron dynamics.

To explain the detailed electronic dynamic behaviours in the near- and below-threshold generation, the positions of the electrons as a function of the time are shown in Fig. 3. Figure 3a presents several short trajectories (first return) and long trajectories (first return and second return) in near-threshold HHG and Fig. 3b indicates the corresponding laser field along with sketches of electron dynamics. For the short trajectories, the electron first tunnels through the lower part of the barrier potential, accelerates and returns to the core. This is a typical tunnelling process. The long trajectory (first return) has a similar mechanism. Nevertheless, when the electron once again returns to the core, it now faces the combined atom-field potential wall (the higher part of the barrier potential, HBP) and tunnelling is unlikely. Thus the electron first moves towards the HBP and subsequently absorbs several photons to a higher energy state and quickly returns to the ground state (second return). Such a mechanism is similar to the multiphoton process.

Figure 3c shows several below-threshold nonresonant short and long trajectories. We find that the electron leaves the core near the zero laser fields and always faces the HBP, where tunnelling ionization is impossible. As a result, the electron is pulled back to the core quickly by the atomic potential, this behaviour only involves the multiphoton process and such a mechanism is demonstrated in Fig. 3d. Note that multi-rescattering is possible in below-threshold short trajectories. Furthermore, we find that the travel time is ∼0.3 optical cycles for both below-threshold short- and long trajectories, but each resonant emission is almost instantaneous, the closer to the peak of the field the more enhanced the harmonic emission (see the SST time-frequency spectra in Fig. 2b). In fact, it is difficult to distinguish below-threshold short and long trajectories, since their dynamical behaviours are similar to the multi-rescattering trajectories in the near-threshold HHG. Figure 3e,f shows several below-threshold resonant trajectories and the corresponding sketches of mechanisms, respectively. The electron leaves the core near the peak of the laser field, and always moves towards the HBP, where tunnelling is unlikely. The electrons absorb several photons and are excited, but quickly return to the core, with a travel time ∼0.1 optical cycle. This multiphoton process contributes only to the resonant trajectories in the below-threshold HHG.

### Dynamical phase of near- and below-threshold HHG

In this section we analyse and uncover the dynamical phase of near- and below-threshold harmonic emission process. According to the time profile for each harmonic^27^, the time at the peak intensities suggest the emission times of such harmonic during the interaction of the atom with the applied field. In addition, the corresponding dynamical phases imply the underlying physical process that gives rise to such harmonic. The time profile 
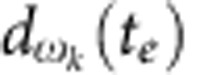
 for a specific harmonic *ω*_*k*_ can be obtained by the reconstruction of the SST representation. The dynamical phase *φ*_*k*_(*t*_*e*_) is therein extracted from 
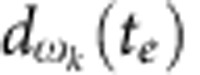
 by 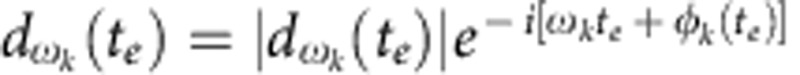.

**Figure 4:**
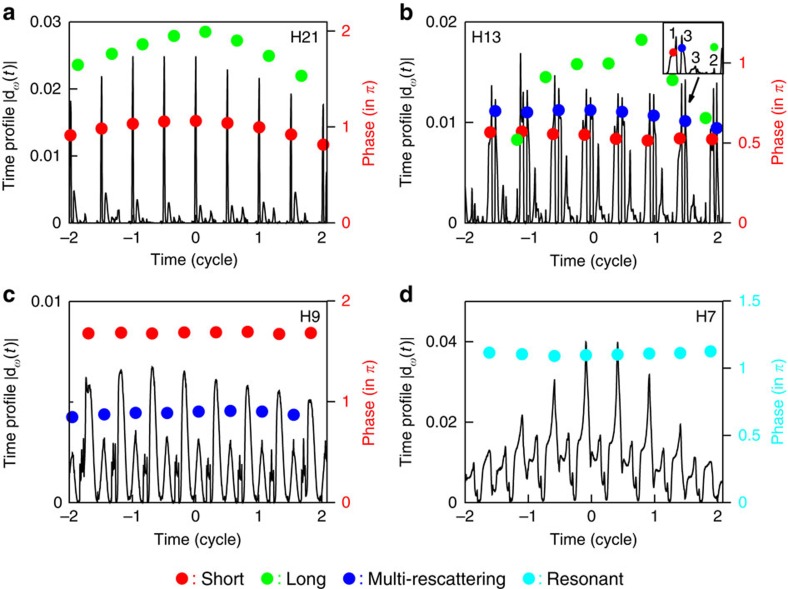
Time profiles and dynamical phase of the harmonic spectra of Cs. (**a**) The time profiles of the 21st harmonic (H21, above threshold). The red dots and green dots are the corresponding dynamical phases for the peak intensity of the short trajectories and long trajectories calculated by the SST, respectively. (**b**) Same as **a**, for the time profiles of the 13th harmonic (H13, near threshold). The blue dots are the corresponding dynamical phases of the multi-rescattering trajectories (second return). The inset shows an enlarged view of the time profiles indicated by the black arrow. Note that the numbers have the same meanings as shown in Fig. 2, namely, short trajectories 1, long trajectories 2, and multi-rescattering trajectories 3 (second return and third return). (**c**) Same as **a**, for the time profiles of the 9th harmonic (H9, below threshold, nonresonant). (**d**) Same as **a**, for the time profiles of the 7th harmonic (H7, below-threshold resonant). The light blue dots are the corresponding dynamical phases of the resonant trajectories. The laser parameters are the same of those in Fig. 1a.

We first consider the time profile and dynamical phases of the 21st harmonic (H21) in Fig. 4a as an example of the harmonics in the above-threshold region, where the tunnelling mechanism is dominant. As shown in the figure, there is a major peak followed by several small peaks within each half optical cycle of the time profile. By marking the dynamical phases of these peaks and comparing the corresponding emission times with those of the semiclassical trajectories in Fig. 2c,d, we find that the major peak corresponds to the short trajectories, while the minor ones correspond to the long trajectories and multi-rescattering trajectories. In addition, the dynamical phase of the long trajectory (green dots) has stronger dependence on the laser intensity than that of the short trajectory (red dots). Similar behaviour is observed in our previous studies of the H-atom^28^, Na-atom^29^ and molecular H_2_ (ref. 30).

Figure 4b shows the time profile of the 13th harmonic (H13), a representative case in the near-threshold regions. The time profile presents a complex pattern within each half optical cycle, suggesting that in addition to the short and long trajectories, the contribution of multi-rescattering trajectories become significant. The inset shows an enlarged view of the time profiles indicated by the black arrow, and the numbers have the same meanings as those in Fig. 2. We find the second return have strong contributions to near-threshold harmonic generation. From our previous study^31^, the contributions of the multi-rescattering trajectories are dominant in the near-threshold region, and the multi-rescattering process depends on the intensity of the laser field. When the laser intensity is increased, the multi-rescattering behaviour of the electrons becomes more pronounced. Fig. 4b also displays the identified dynamical phases for the short (red dots) and long trajectories (green dots), along with those for the largest multi-rescattering trajectories (blue dots, second return). Similar to the results in H21, the dynamical phases for the long trajectories are sensitive to the laser profile, whereas those for the short trajectories are not. As for the largest multi-rescattering trajectories, the corresponding dynamical phases are almost constant during the emission process. The phase invariance leads to a zero chirp, thereby implying the multiphoton process is dominant, as explained in Fig. 3a.

Figure 4c shows the time profile of the 9th harmonic (H9). Within each half optical cycle, the dominant peak is recognized as the short trajectory, despite its large phase (located between 1.5–2 *π*). Furthermore, the multi-rescattering trajectories still contribute to the generation of the harmonics in this region. The time profile for the 7th resonant harmonic (H7) is shown in Fig. 4d. We observe that the envelope of the time profile resembles that of the laser profile. In the multiphoton regime, the probability of absorbing *N* photons is roughly proportional to the *I*^*N*^(*t; I* is the laser intensity)^23^. The major peaks are indicated as the resonant trajectories and their dynamical phases are denoted by light blue dots. Since the influence of the multi-rescattering trajectories becomes smaller, the harmonic generated by the multiphoton resonant harmonic is composed of mainly a single phase, indicating that the dynamical phases are locked.

## Discussion

We have presented an *ab initio* study of on the near- and below-threshold harmonic generation of Cs atoms in an intense mid-infrared laser fields by accurately solving the TDSE. An accurate angular momentum-dependent model potential is constructed for the description of the Cs atom. We have performed the quantum trajectories analysis of the near- and below-threshold harmonics by using the SST time-frequency profiles and identified the trajectories with an extended semiclassical simulation. We find that multiphoton-dominated short trajectories, long trajectories, multi-rescattering trajectories and resonant trajectories in the near- and below-threshold HHG involve only the electron scattered off the combined atom-field potential wall followed by the absorption of photons. In particular, we find that only the resonant trajectories possess the single phase-component, which implies that the dynamical phases in below-threshold resonance harmonic generation are locked. Furthermore, we find that the near-threshold harmonic generation represents a complex physical processes where all kinds of the trajectories contribute to HHG. However, the effects of multi-rescattering trajectories gradually disappear in the lower-order harmonic generation. Our result enables us to obtain a deeper understanding of the mechanism of near- and below-threshold harmonic generation and provides an insight into the field of ultrafast science and technology.

## Methods

### Time-dependent Schrödinger equation

The HHG power spectra can be investigated accurately and efficiently by solving the TDSE in space and time by means of the time-dependent generalized pseudospectral method^21^. To obtain the accurate calculation of the harmonic spectra of Cs, an angular momentum-dependent model potential is constructed in the following form:


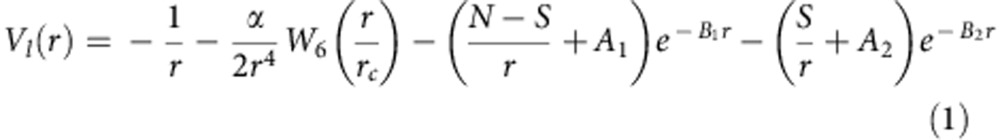


where *α* is the Cs^+^ core dipole polarizability, *W*_6_ is a core cutoff function and *r*_c_ is an effective Cs^+^ core radius.

In the present work, we find it is sufficient to use two different angular momentum-dependent model potential, one for the states with *l*=0 and another for the states with *l*⩾1. The values of the parameters determined are listed in Table 1. In addition, we present a comparison of the bound-state energies predicted by this model potential and the experimental values(Supplementary Table 1). The agreement is very good in all the cases.

Once the time-dependent wave function is available, we can calculate the expectation value of the induced dipole moment, and the HHG power spectra can be obtained by the Fourier transform of the time-dependent dipole moment.

### SST time-frequency analysis

In this work, we perform the time-frequency analysis of the harmonic spectra of Cs by means of SST method. The SST is proposed to address the intrinsic blurring in the linear type time-frequency methods, such as the Gabor transform and Wavelet transform, and the accuracy and limitation of the SST technique are well supported by mathematical analysis.

### Semiclassical method

To understand the quantum-trajectory characteristics, we have extended the semiclassical three-step model by including the accurate electronic structure information in the selective analysis of the quantum trajectories in the near- and below-threshold generation of Cs. The initial condition is that the electrons are released at the core with the initial velocity along or opposite the polarization direction of the laser field. In our calculation, the semiclassical results are obtained by solving the Newton's equation including the atomic potential, which is given by


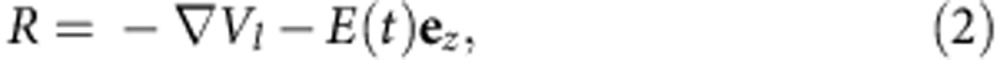


where *E*(*t*) is the electric field of the laser pulse and *V*_*l*_ is the angular momentum-dependent model potential of Cs atoms.

## Additional information

**How to cite this article**: Li, P.-C. *et al*. Dynamical origin of near- and below-threshold harmonic generation of Cs in an intense mid-infrared laser field. *Nat. Commun.* 6:7178 doi: 10.1038/ncomms8178 (2015).

## Supplementary Material

Supplementary Information Supplementary Figure 1, Supplementary Table 1, Supplementary Methods and Supplementary References

## Figures and Tables

**Table 1:** Model potential parameters for Cs (in a.u.).

**l**	**α**	***r***_***c***_	***S***	***N***	***A***_**1**_	***A***_**2**_	***B***_**1**_	***B***_**2**_
0	15.6	3.35	59.151687	54	32.3293670	0	1.69	4.67
⩾1	15.6	3.35	41.035779	54	0.32427851	30	1.20	5.00

a.u., arbitrary units.
