# Supplementary material for: Dynamical origin of near- and below-threshold harmonic generation of Cs in an intense mid-infrared laser field
Source: Nat Commun. 2015 May 20;6:7178. doi: 10.1038/ncomms8178 (PMC4455065; doi:10.1038/ncomms8178)
Supplement: Supplementary Information — Supplementary Figure 1, Supplementary Table 1, Supplementary Methods and Supplementary References [file ncomms8178-s1.pdf]

Supplementary Figures

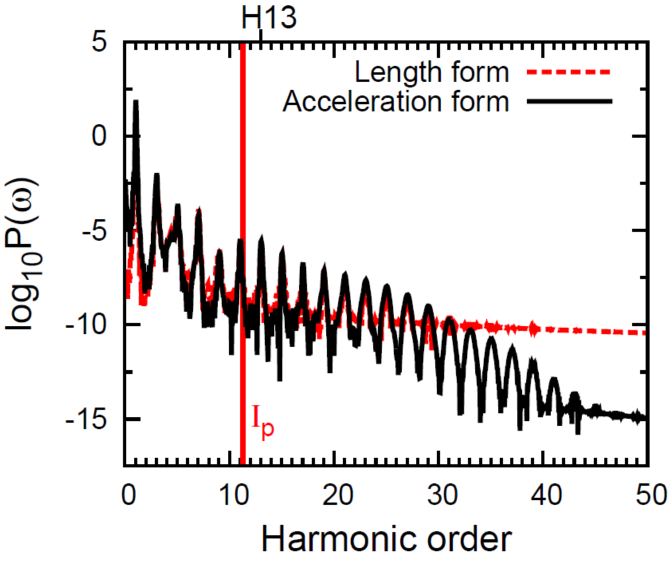

Supplementary Figure 1: The high-order harmonic generation (HHG) power spectrum of Cs in the length (red dashed line) and acceleration (black solid line) forms driven by a mid-infrared 3600-nm laser pulse. The red solid line indicates the corresponding ionization threshold marked by I_p.

Supplementary Tables

Supplementary Table 1: Comparison of the calculated atomic Cs energies with the experimental values (in a.u.). For each angular momentum l, two rows of energies $E_{n,l}$ are listed: the first row refers to the calculated model-potential energies, and the second row refers to the experimental values[1].

	Energy $E_{n,l}$			
n	$l=0$	$l=1$	$l=2$	$l=3$
4				-0.0316125
				-0.0315950
5			-0.0767537	-0.0202222
			-0.0767681	-0.0202083
6	-0.1430990	-0.0904542	-0.0401010	-0.0140292
	-0.1430990	-0.0904751	-0.0400590	-0.0140198
7	-0.0586446	-0.0434199	-0.0243948	-0.0102970
	-0.0586446	-0.0433755	-0.0243585	-0.0102905
8	-0.0323019	-0.0257369	-0.0163741	-0.0078767
	-0.0323015	-0.0257080	-0.0163492	-0.0078722
9	-0.0204845	-0.0170564	-0.0117426	-0.0062189
	-0.0204845	-0.0170386	-0.0117258	-0.0062156
10	-0.0141531	-0.0121389	-0.0088300	-0.0050341
	-0.0141531	-0.0121274	-0.0088183	-0.0050316

Supplementary Methods

1. *Ab initio* Simulation of the High-order Harmonic Generation Spectra of Cs

In the length gauge, the TDSE in the dipole approximation for an atom interacting with a laser field is given by,

$$i\frac{\partial\psi(\mathbf{r},t)}{\partial t} = \left[\widehat{H}_0 + \widehat{V}(\mathbf{r},t)\right]\psi(\mathbf{r},t), \tag{1}$$

where $\widehat{V}(\mathbf{r},t)$ is the time-dependent atom-field interaction, and $\widehat{H}_0$ represents unperturbed atom Hamiltonian. $\widehat{H}_0$ is given as

$$\widehat{H}_0 = -\frac{1}{2}\nabla^2 + \sum_l |\,Y_l^0 > V_l < Y_l^0|, \tag{2}$$

where V_l is the model potential of atomic Cs for each angular momentum l, and Y_l^0 is the spherical harmonic.

To obtain the accurate calculation of the harmonic spectra of Cs, an angular-momentum-dependent model potential is constructed as the following form:

$$V_l = -\frac{1}{r} - \frac{\alpha}{2r^4}W_6\left(\frac{r}{r_c}\right) - \left(\frac{N-S}{r} + A_1\right)e^{-B_1 r} - \left(\frac{S}{r} + A_2\right)e^{-B_2 r}, \tag{3}$$

where α is the Cs^+ core dipole polarizability, W_6 is a core cutoff function [2,3] given by

$$W_n(x) = 1 - [1 + nx + \frac{(nx)^2}{2!} + \cdots + \frac{(nx)^n}{n!}]e^{-nx}, \tag{4}$$

and r_c is an effective Cs^+ core radius.

In the present work we find it is sufficient to use two different angular-momentum-dependent model potentials, one for states with l and another for states with $l \geq 1$. Supplementary Table I presents a comparison between the bound-state energies predicted by this model potential and the experimental values. The two values are in good agreement.

The TDSE is solved accurately and efficiently by means of the time-dependent generalized pseudospectral method (TDGPS)[4]. Once the time-dependent wave function $\psi(\mathbf{r}, t)$ is available, we can calculate the expectation value of the induced dipole moment in the length and acceleration forms, respectively,

$$d_L(t) = < \psi(\mathbf{r}, t)|z|\psi(\mathbf{r}, t) >, \tag{5}$$

$$\begin{aligned} d_A(t) &= \frac{\partial^2}{\partial t^2} < \psi(\mathbf{r}, t)|z|\psi(\mathbf{r}, t) > \\ &= -< \psi(\mathbf{r}, t)|[\hat{H}, [\hat{H}, z]]|\psi(\mathbf{r}, t) >. \end{aligned} \tag{6}$$

The high-order harmonic generation (HHG) power spectra in the length and acceleration forms can be obtained by the Fourier transformation of time-dependent dipole moment $d_L(t)$ and $d_A(t)$[2], respectively,

$$P_L(\omega) = |\frac{1}{t_f - t_i} \int_{t_i}^{t_f} d_L(t) e^{-i\omega t} dt|^2, \tag{7}$$

$$P_A(\omega) = |\frac{1}{(t_f - t_i)\omega^2} \int_{t_i}^{t_f} d_A(t) e^{-i\omega t} dt|^2. \tag{8}$$

Supplementary Figure 1 shows the length-form and acceleration-form HHG power spectrum of atomic Cs described by the angular-momentum-dependent model potential in mid-infrared laser field. In calculation, we adopt the same 3600-nm mid-infrared laser pulse with a cosine-squared shape profile, a duration of 20 optical cycles, and an intensity of $I = 1.4 \times 10^{12}$ Wcm^{-2}. In Supplementary Fig. 1 the whole range spectra of the length and acceleration forms are nearly identical. For each harmonic above the ionization threshold, both forms present the same detail structures. Beyond the cut-off region, while the length form spectrum levels off, the acceleration form spectrum reveals more harmonics.

2. Synchrosqueezing Transform

We perform the time-frequency analysis on the induced dipole moment $d(x)$ of atomic Cs interacting with the applied laser field by means of the synchrosqueezing transform (SST)[5-7]. The SST is described as:

$$S(t, \xi) = \int \frac{1}{\sqrt{\omega}} V(t, \omega) \frac{1}{\alpha\sqrt{\pi}} e^{-\left(\frac{\xi - \Omega_f(t,\omega)}{\alpha}\right)^2} \mathrm{d}\omega \,, \tag{9}$$

where $V(t, \omega)$ is the Morlet wavelet transform, $\Omega_f(t, \omega)$ is the reallocation rule function, and α is a smoothing parameter. In this study, $\alpha = 2.6$. The Morlet wavelet transform is given as:

$$V(t,\omega) = \int d(x)\sqrt{\omega}W(\omega(x-t))\mathrm{d}x\ , \tag{10}$$

where

$$W(\zeta) = \frac{1}{\sqrt{\tau}}e^{i\zeta}e^{-\frac{\zeta^2}{2\tau^2}} \tag{11}$$

is the mother wavelet. The reallocation rule function is defined as:

$$\Omega_f(t,\omega) = \begin{cases} \frac{-i\partial_t V(t,\omega)}{V(t,\omega)} & for\ V(t,\omega) \neq 0 \\ \infty & for\ V(t,\omega) = 0 \end{cases}\ , \tag{12}$$

where ∂_t denotes the partial derivative in the temporal axis.

The time profile $d_{\omega_k}(t_e)$ for some harmonic ω_k from the SST analysis can be obtained from the reconstruction function:

$$d_{\omega_k}(t_e) = \Re e\left\{R_W^{-1}\int_{\xi_1}^{\xi_2} S(t,\xi)\frac{1}{\sqrt{\omega}}\mathrm{d}\omega\right\}, \tag{13}$$

where (ξ_1, ξ_2) is the neighborhood of such harmonic, $R_W = \int \frac{\widehat{W}(\eta)}{\eta}\mathrm{d}\eta$ and $\widehat{W}(\eta)$ is the Fourier transform of $W(\zeta)$, and $\Re e$ denotes the real part.

Supplementary References

1. Kramida, A., Ralchenko, Yu., Reader, J., & NIST ASD Team (2013). NIST Atomic Spectra Database (ver. 5.1), [Online]. Available: http://physics.nist.gov/asd [2013, December 25]. National Institute of Standards and Technology, Gaithersburg, MD.

2. Chu, X. & Chu, S.I., Laughlin, Cecil. Spectral and temporal structures of high-order harmonic generation of Na in intense mid-ir laser fields. *Phys. Rev. A* **64**, 013406 (2001).

3. Li, P. C., Laughlin, Cecil., & Chu, S.I. Generation of isolated sub-20-attosecond pulses from He atoms by two-color midinfrared laser fields. *Phys. Rev. A* **89**, 023431 (2014).

4. Tong, X. M., & Chu, S. I. Theoretical study of multiple high-order harmonic generation by intense ultrashort pulsed laser fields: A new generalized pseudospectral time-dependent method. *Chem. Phys.* **217**, 119 (1997).

5. Daubechies, I., Lu, J., & Wu, H. T. Synchrosqueezed wavelet transforms: an empirical mode decomposition. *Appl. Comput. Harmon. Anal.* **30**, 2 (2011).

6. Chen, Y. C., Cheng, M. Y., & Wu, H. T. Non - parametric and adaptive modelling of dynamic periodicity and trend with heteroscedastic and dependent errors. *J. R. Stat. Soc. Ser. B Stat. Methodol.* **76**, 3(2014).

7. Sheu, Y. L., Hsu, L. Y., Wu, H. T., Li, P. C., & Chu, S.I. A new time-frequency method to reveal quantum dynamics of atomic hydrogen in intense laser pulses: Synchrosqueezing transform. *AIP Advances* **4**, 117138 (2014).
